# Elucidation of the sequential transcriptional activity in Escherichia coli using time-series RNA-seq data

**DOI:** 10.6026/97320630013025

**Published:** 2017-01-31

**Authors:** Pui Shan Wong, Kosuke Tashiro, Satoru Kuhara, Sachiyo Aburatani

**Affiliations:** 1 Biotechnology Research Institute for Drug Discovery, National Institute of AIST, Tokyo, Japan;; 2 Graduate School of Bioresource and Bioenvironmental Sciences, Kyushu University, Fukuoka, Japan;; 3 Com. Bio Big Data Open Innovation Lab. (CBBD-OIL), National Institute of AIST, Tokyo, Japan;

**Keywords:** Escherichia coli, gene regulation, network, time-series

## Abstract

Functional genomics and gene regulation inference has readily expanded our knowledge and understanding of gene interactions with regards to expression regulation. With the advancement of transcriptome sequencing in time-series comes the ability to study the sequential changes of the transcriptome. Here, we present a new method to augment regulation networks accumulated in literature with transcriptome data gathered from time-series experiments to construct a sequential representation of transcription factor activity. We apply our method on a time-series RNA-Seq data set of Escherichia coli as it transitions from growth to stationary phase over five hours and investigate the various activity in gene regulation process by taking advantage of the correlation between regulatory gene pairs to examine their activity on a dynamic network. We analyse the changes in metabolic activity of the pagP gene and associated transcription factors during phase transition, and visualize the sequential transcriptional activity to describe the change in metabolic pathway activity originating from the pagP transcription factor, phoP. We observe a shift from amino acid and nucleic acid metabolism, to energy metabolism during the transition to stationary phase in E. coli.

## Background

After mapping out a genome, the study of gene regulation processes and their effect on gene expression is generally the next stage in the analysis pipeline and is an important topic in system biology. It is a challenging topic due to the way it interconnects components in the genome, the proteome and the epigenome as they contribute to the control of the magnitude, location and timing of gene expression [1,2]. Generally, the solution to the challenge is a functional genomics approach whenever it is possible. Since functional genomics methods rely on a set of known inducers for transcription factors, model organisms such as Escherichia coli are the main candidates [3].

A more accessible approach comes from reverse engineering gene regulation links from transcriptome data using statistical models and algorithms that can rely on observed measurements of transcripts alone or with the inclusion of non-transcriptome data like protein concentration [4]. As more transcriptome data has become available, the development of analysis methods have adapted accordingly and there are now a variety of methods utilizing different statistical models such as ANOVA in ANOVerence [5] and mutual information measures in ARACNE [6]. There has also been a development in our understanding of different gene regulation components but analysis tools have been able to continue to account for and include new regulation mechanisms. To make sense of the data despite the complexity of the underlying processes, studies began including meta-analyses to the gene regulation analysis pipeline where the method involved combining results from different models more robust summary statistics [7], leading to collaborative efforts to integrate different models.

The next step in gene regulation research lies within timeseries data which has an advantage over a single time point treatment and control experiment because it can detect patterns of gene expression over time such as periodic patterns in response to stimulus [8]. This type of data was challenging to gather using a functional genomics approach but the advancement of high throughput sequencing made it more practical to perform experiments that involve taking samples at many time points over a long period. Such data need the development of appropriate analysis methods to extract relevant biological information in a suitable amount of time [9].

To elucidate the dynamics of time-series gene regulation, we present a method that uses the cross-correlation between transcription factor and gene expression for efficient analysis of numerous genes. We use cross-correlation in place of correlation as correlation is not able to capture the sequential changes in gene expression that exist in time-series data, while the use of differential equations is difficult to apply to large data sets. The relationship between the expression of the regulating transcription factors and the genes they regulate is used to identify crucial times of activity in order to build a sequence of regulation events. These activation times come in pairs when the gene expressions of transcription factors change with a correspondingly similar change in gene expression in the genes they regulate. We apply our method to the E. coli model as it provides a solid foundation with established literature and produces fuller networks, and then show the gene regulation activity for the pagP gene and its associated regulators.

## Methodology

The expression data was normalized in R [10] using DESeq [11] and then log transformed. The time points used in this analysis were 3, 4, 5, 6 and 8 hours. The three biological samples were analysed separately and genes which were not expressed at all were excluded from the rest of the analysis.

### Regulatory Activation Time

A transcription factor a and the gene it regulates b form a regulatory pair. The time when a initiated its activity ta and the time when the expression of b responded tb is calculated per pair. Genes with only one transcript configuration were selected and self-regulating genes with no other regulators in its regulatory process were excluded.

**Figure 1:**
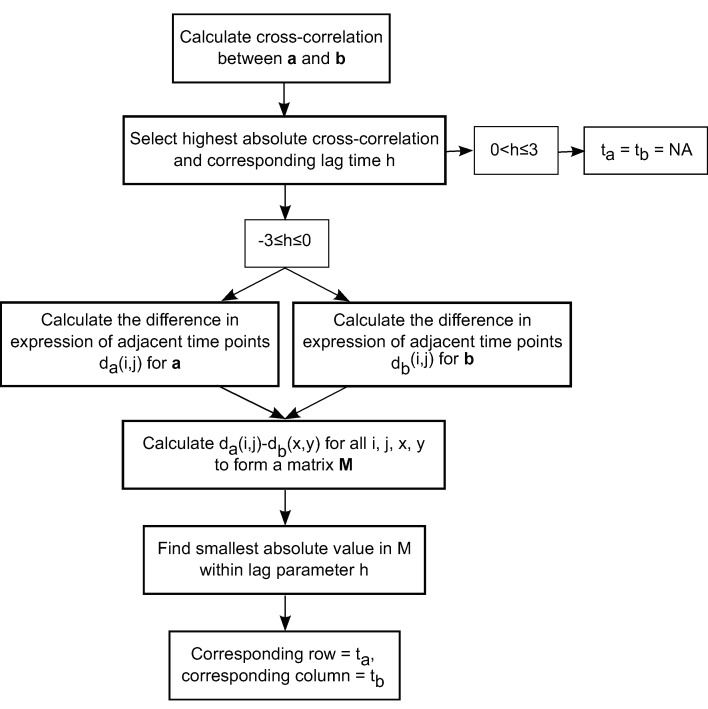
A flow chart of the lag time h detection method starting from top to bottom. The cross-correlation between a transcription factor a and regulated gene b is first calculated and the highest cross-correlation is selected. The corresponding lag time h of the highest cross-correlation is used to exclude the a and b pair if 0 < h ≤ 3 otherwise the analysis continues. The difference in expression of adjacent time points are calculated for a and b individually, and then the difference between those differences are calculated in a matrix, M where da were the rows and db were the columns. The activation times ta and tb are defined as the row and column of the smallest absolute value in M.

**Figure 2:**
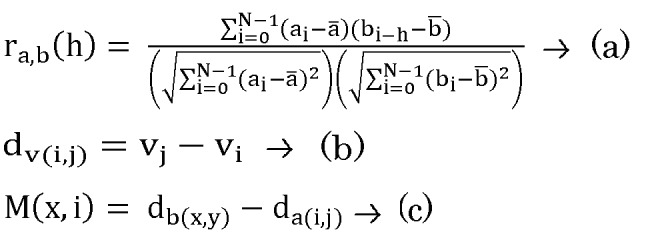
The three equations used in the methodology for network construction. (a) The calculation of cross-correlation between transcription factor a and regulated gene b for lag time h for 0 ≤ i + h < N where N is the number of values in a and b, and a¯ and ¯b are their respective means. (b) The difference in expression between adjacent time points i and j where i and j are either 3, 4, 5, 6, 8 and j > i, for a gene v (c) A matrix M populated by pairwise differences between all combinations of da(i,j) and db(x,y) so that da were ordered along the rows and db were ordered along the columns.

The lag time h was determined by calculating the crosscorrelation between the transcription factor a and regulated gene b for all possible h (Figure 2.(a)). Regulator pairs with lag time 0 < h ≤ 3 were discarded. The remaining pairs were −3 ≤ h ≤ 0 so that ta ≥ tb . The largest absolute crosscorrelation |ra,b (h)| was chosen to select the lag time h (Figure 1). The activation times dv(i,j) are based on the difference in expression between adjacent time points i and j where i and j are either 3, 4, 5, 6, 8 and j > i, for a gene v (Figure 2.(b)). The pairwise differences between all combinations of da(i,j) and db(x,y) were inserted into a matrix M where da were ordered along the rows and db were ordered along the columns (Figure 2.(c)). The smallest absolute value among the eligible numbers was selected and the activation times ta and tb were assigned to the matching x and i.

### Visualization

A network was created using the igraph package [12] so that transcription factors and regulated genes were rendered as vertices and the regulation relations between them were rendered as edges. The edges were directed from transcription factors to regulated genes and loops were allowed. Single vertices were excluded as both transcription factor and regulated gene must be present in the network.

The network was converted into a dynamic network using the networkDynamic [13] and ndtv [14] packages. The activation times were added so vertices were coloured green at activation time, ta and tb for the respective vertex, and edges were coloured starting from the activation time of the transcription factor ta until the activation time of the regulated gene tb .

### Data Source

The E. coli b str. REL606 expression data from [15] is available at the Dryad Digital Repository: http://dx.doi.org/10.5061/dryad.hj6mr. The genome information for E. coli b str. REL606 was downloaded from Genbank (NC 012967) [16] and the identifiers were cross referenced with E. coli K-12 using the cross referencing tool at Ecogene [17]. The gene regulation information was downloaded from RegulonDB [18] and the metabolic pathway information was downloaded from KEGGscape [19]. Further cross referencing data was downloaded using AnnotationHub [20].

## Results and Discussion

We propose a gene expression regulation analysis approach to detect the times at which gene expression regulators initiate a change and the times at which the genes being regulated have their expressions altered. These times create a sequential activity map of transcription factors and genes that describe the pattern of metabolic reactions during a given period of time. The method was applied on experimental time-series data from E. coli over a period of five hours and the regulation process involving phoP and pagP activity was used to show how the results could be visualized and interpreted in a dynamic network.

Time-series experiments are different from how static experiments are investigated so there was an initial investigation which found that cross-correlation was better fitted to the timeseries data compared to correlation between the expression of a transcription factor and regulated gene. Pearson correlation was 0.0709 overall and -0.506 for average negative correlation and 0.556 for average positive correlation while in contrast, crosscorrelation values were -0.738 for average negative correlation and 0.766 for average positive correlation. By allowing for time lag, we were better able to identify regulation events, even though there was still some variability that could not be accounted for with cross-correlation alone.

### Time Detection

The chosen lag times were used to filter out the possible transcription factor and gene activation times. The most common combination of activity time in sample 1 and 2 is 3 x 3 hours which is a lag time of 0 (Table 1). Sample 3 was at 4 x 4 hours but the 3 x 3 hour combination was the next most frequent which is still a lag time of 0. The most common active time for genes were 5 hours in sample 1 and 2, and 6 hours in sample 3. The one hour difference was consistent between the most common transcription factor activation time and gene activation time. Combinations of transcriptome and metabolome analyses have detected some variation of time lag in E. coli gene regulation previously, with some studies detecting a common time lag of one hour [21] while others fall between 10 and 20 minutes [22]. The higher frequency of 0 or -1 lag time counts across all samples for the pairs in the analysis concurred with some of these studies. For the 0 lag time pairs that were detected, they may have shorter lag times than the sampling time, such as 10 or 20 minutes, so were not detected in our data set.

The three samples in the data set were analysed separately to see if there would be a consensus result between them. The expression of each time point per sample was highly correlated with each other which indicated a sufficient degree of similarity between them for direct comparison (between 0.912 and 0.709). When inspecting the range of detected active times, we observed a general agreement of lag times between the samples. Although the actual lag times and active times differed, sample 3 seemed to be an hour behind samples 1 and 2 metabolically. This difference in time was present in the majority of genes analysed and suggested sampling or biological error. The consistency seen in the results indicate that the length of the time period between transcription factor action and gene expression change was more relevant than the gene expression value itself, and that differences between samples can be detected in this way.

### Network Visualization

**Figure 3:**
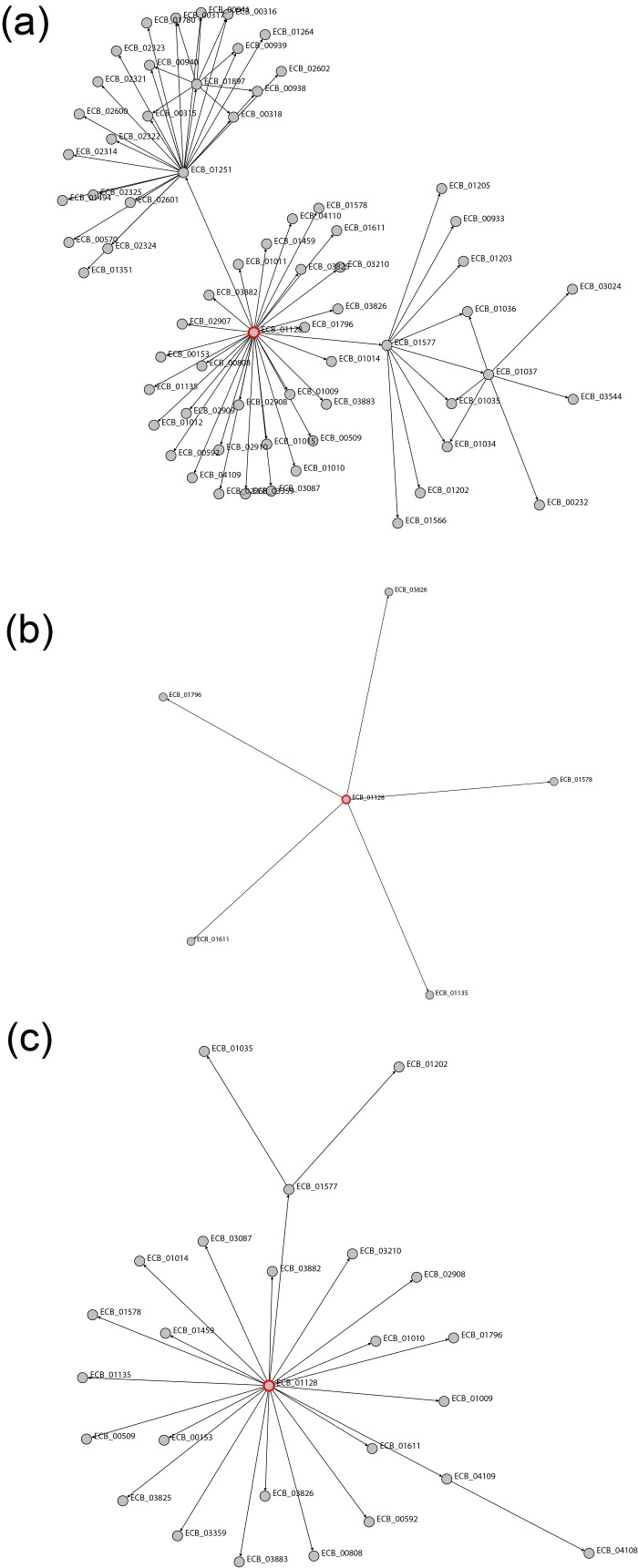
The three networks created by each sample with phoP highlighted in red. The dynamic highlights of activation time and labels, and visualization controls are not shown. (a) Sample 1 produced the largest network containing 67 vertices and 80 edges, (b) sample 2 contained 6 vertices and 6 edges and (c) sample 3 contained 26 vertices and 26 edges.

The network produced from sample 1 data (Figure 3(a)) was 67 vertices and 80 edges in size. It contained 5 regulators, which were either part of the two-component system or had no metabolic pathway information. They were phoP, cysB, cbl, rstA and csgD. The remaining vertices were genes that, as a group, were members of 24 unique pathways. In the early half of the experiment, activated genes were members of various amino acid pathways such as histidine and lysine metabolism with a few genes in ABC transporters and one carbon pool by folate pathways. Nucleotide pathways were present between 3 and 6 hours, and absent during the 6 to 8 hour period. In the latter half of the experiment, there were more activated gene activity related to energy metabolism such as methane, nitrogen and sulfur metabolism.

The network produced from sample 2 data (Figure 3(b)) was the smallest of all samples with 6 vertices and 6 edges, and only had a few active regulators and genes. The activation times were at 3 or 5 hours and the activation times for the transcription factor and regulated gene was the same for each pair. There were two genes with pathway annotations and pagP was not present as it had no detected activation time in this sample. The two genes with pathway annotations were rstB and metL which were part of the twocomponent system, biosynthesis of secondary metabolites and microbial metabolism in diverse environments.

The network produced from sample 3 (Figure 3(c)) was moderate in size containing 26 vertices and 26 edges. The regulators were all part of the two- component system or had no pathway information while the genes were consistently involved in the biosynthesis of secondary metabolites or microbial metabolism in diverse environments through all time points. Genes that were activated earlier tended to be part of pathways related to amino acid metabolism such as cysteine and methionine metabolism, while genes activated later tended to be part of pathways such as sulfur metabolism.

The pagP gene was previously identified as a principal element of phase transition [15] and it is regulated by the phoP transcription factor. The separate analysis of the three samples was consolidated with the construction of the dynamic networks. Analysis of each sample successfully led to the assembly of networks from which we were able to identify and isolate processes related to the phoP transcription factor. The network created from sample 2 was the consensus structure that was found in the other two networks, indicating that the regulatory activity that it contained is central to stationary phase transition. The phoB P binding sites are thought to be involved in the timing of transcription in phosphate assimilation genes [23] and our observations seem to concur with that since the initiation of phoP occurred early in all samples. The compilation of metabolic pathway activity in all the networks indicated that phoP activity influenced the metabolic activity of amino acid metabolism from high to low as the E. coli transitioned into stationary phase and at the same time, the increase in metabolic activity of different types of energy metabolism. Since binding affinity is related to response time of expression activity, this suggests further research on the promoters at the outer edges of the network is a possibility [24].

## Conclusion

Our method for isolating the times at which transcription factors initiate gene regulation and when the change in gene expression starts was successful and the results were complemented by a dynamic network visualization. We were able to show the sequential regulation activity initiated by phoP on the genes it directly regulated as well as genes further downstream of its activity. The network showed a small part of the ripple effect that transcription factors can have on regulation systems in a given time period. The visualization identified the types of metabolic pathways that were activated and deactivated as E. coli reaches stationary phase and determined the time and sequence of activities. Although this was used on a simplified data set, it is possible to extend the analysis to other types of experiments as a type of regulation profile of metabolic processes.

## Figures and Tables

**Table 1:** Frequency counts of activation times ta and tb for transcription factor a and the gene it regulates b.

	Transcription Factor a (hr)					
	Gene b (hr)	3	4	5	6	Sum
Sample 1	3	650				650
	4	130	491			621
	5	156	82	539		777
	6	155	177	99	138	569
	Sum	1,091	750	638	138	2,617
Sample 2	3	571				571
	4	77	197			274
	5	172	118	286		576
	6	37	219	89	212	557
	Sum	857	534	375	212	1,978
Sample 3	3	299				299
	4	285	497			787
	5	141	235	297		673
	6	160	218	184	191	753
	Sum	885	950	481	191	2,507
